# Toward clearer recognition and easier usefulness: development of a cross-lingual atherosclerotic cerebrovascular disease ontology

**DOI:** 10.1093/database/baae117

**Published:** 2024-12-05

**Authors:** Hetong Ma, Liu Shen, Jiayang Wang, Shilong Wang, Min Wang, Meng Wang, Zixiao Li, Jiao Li

**Affiliations:** Intelligent Computing Department, Institute of Medical Information & Library, Chinese Academy of Medical Sciences/Peking Union Medical College, No. 3 Yabao Road, Beijing 100020, China; Intelligent Computing Department, Institute of Medical Information & Library, Chinese Academy of Medical Sciences/Peking Union Medical College, No. 3 Yabao Road, Beijing 100020, China; Intelligent Computing Department, Chinese Academy of Medical Sciences/Peking Union Medical College, No. 3 Yabao Road, Beijing 100020, China; Computer Science Department, Harbin Institute of Technology, No. 92, Xidazhi Street, Harbin 150001, China; Intelligent Computing Department, Institute of Medical Information & Library, Chinese Academy of Medical Sciences/Peking Union Medical College, No. 3 Yabao Road, Beijing 100020, China; Department of Neurology, Beijing Tiantan Hospital, Capital Medical University, No. 119 South Fourth Ring West Road, Beijing 100070, China; China National Clinical Research Center for Neurological Diseases, Beijing Tiantan Hospital, Capital Medical University, No. 119 South Fourth Ring West Road, Beijing 100070, China; National Center for Healthcare Quality Management in Neurological Diseases, Beijing Tiantan Hospital, Capital Medical University, No. 119 South Fourth Ring West Road, Beijing 100070, China; Department of Neurology, Beijing Tiantan Hospital, Capital Medical University, No. 119 South Fourth Ring West Road, Beijing 100070, China; China National Clinical Research Center for Neurological Diseases, Beijing Tiantan Hospital, Capital Medical University, No. 119 South Fourth Ring West Road, Beijing 100070, China; National Center for Healthcare Quality Management in Neurological Diseases, Beijing Tiantan Hospital, Capital Medical University, No. 119 South Fourth Ring West Road, Beijing 100070, China; Research Unit of Artificial Intelligence in Cerebrovascular Disease, Chinese Academy of Medical Sciences, Beijing Tiantan Hospital, Capital Medical University, No. 119 South Fourth Ring West Road, Beijing 100070, China; Chinese Institute for Brain Research, No. 9 Yike Road, Beijing 102206, China; Intelligent Computing Department, Institute of Medical Information & Library, Chinese Academy of Medical Sciences/Peking Union Medical College, No. 3 Yabao Road, Beijing 100020, China

## Abstract

Atherosclerotic cerebrovascular disease could result in a great number of deaths and disabilities. However, it did not acquire enough attention. Less information, statistics, or data on the disease has been revealed. Thus, no systematic concept datasets were released to help clinicians clarify the scope, assist research, and offer maximized value. This study aimed to develop a cross-lingual atherosclerotic cerebrovascular disease ontology; describe the workflow, schema, hierarchical structure, and the highlighted content; design a brand-new rehabilitation ontology; implement the ontology evaluation; and illustrate the application scenarios in real-world scenarios. We implemented nine steps based on the Ontology Development 101 methodologies combined with expert opinions. The ontology included collection and specification of clinical requirements, background investigation and knowledge acquisition, ontology selection and reuse, scope identification, schema definition, concept extraction, concept extension, ontology verification, and ontology evaluation. We evaluated the proposed ontology in the literature classification task. The current ontology included 10 top-level classes, respectively, clinical manifestation, comorbidity, complication, diagnosis, model of atherosclerotic cerebrovascular disease, pathogenesis, prevention, rehabilitation, risk factor, and treatment. There are 1715 concepts in the 11-level ontology, covering 4588 Chinese terms, 6617 English terms, and 972 definitions. The ontology could be applied in real-world scenarios such as information retrieval, new expression discovery, named entity recognition, and knowledge fusion, and the use case proved that it could offer satisfying support to related medical scenarios. The ontology was proven to be useful in text classification tasks, and the weight-*F*1 score could reach >80% combined with the pretrained model. The proposed ontology provided a clear set of cross-lingual concepts and terms with an explicit hierarchical structure, helping scientific researchers to quickly retrieve relevant medical literature, assisting data scientists to efficiently identify relevant contents in electronic health records, and providing a clear domain framework for academic reference.

**Database URL**: https://bioportal.bioontology.org/ontologies/ACVD_ONTOLOGY

## Introduction

The atherosclerotic cerebrovascular disease could lead to the most ischemic strokes and the following death and disability [1]. One previous study defined it as the aggregation of “ischemic stroke and transient ischemic attack” [2]. Despite the importance and the great harm of the atherosclerotic cerebrovascular disease, less evidence and data were revealed for the atherosclerotic cerebrovascular disease. Information could, to some extent, be extracted from a closely related disease, such as stroke. A study showed that ischemic cerebrovascular disease constituted the majority of strokes, occupying 60%–80% [3, 4]. About 795 000 people suffered from a stroke every year in the USA, and more than three-quarters of them were first-time strokes, while others otherwise experienced recurrent ones [5, 6]. The stroke would result in neurological disorders, responsible for 5.5 million deaths and about 116 million global disability-adjusted life-years lost in 2016 [7–11]. In China, the first-time stroke prevalence was 297 per 100 000 people, >2.5 million new stroke patients would emerge each year, and about three-quarters of them were not able to take care of themselves [4, 12]. Ischemic stroke is mainly distributed in a series of subtypes: cardioembolism, large artery atherosclerosis, small vessel occlusion, other determined causes, and undetermined causes [13]. Stroke rehabilitation was a crucial component of continuity in stroke care [14]. Rehabilitation aims at assisting people with stroke or other disabled situations to recover from poor physical condition, restart body activities, enhance self-care ability, and promote the combination with family and community [15]. Rehabilitation is an indispensable part of stroke treatment and should be paid more and more attention. The best approach targeting stroke recovery is intensive rehabilitation and it should be initiated immediately once the diagnosis is determined with feasibility [16, 17]. The functionality could be promoted higher if patients commenced their rehabilitation during the acute phase [18, 19]. Concentrated rehabilitation training could enhance body function, improve living quality, implement complication prevention, and ease the family burden [17, 20]. During the investigation into stroke rehabilitation, only ∼12% literature on the topic of stroke mentioned rehabilitation. The rehabilitation should be paid much more attention, and more work should be completed toward rehabilitation.

Considering the importance of atherosclerotic cerebrovascular disease, which led to inconvenience for researchers to use or refer to, an ontology could be a great way to help researchers to know, discuss, retrieve, and reuse the concepts in that field, which is defined as “an explicit specification of a conceptualization” [21], namely a set of terms or vocabularies in a specific domain including relevant classes, relations, and rules [22]. A medical ontology could collect information in various ways, display well-organized and explicit knowledge, and provide generation via ratiocination [22]. We investigated atherosclerotic cerebrovascular disease-related ontologies and terminologies such as the stroke ontology (STO) [23], the neurological disease ontology [24], symptom ontology [25], the human phenotype ontology (HPO) [26], the diagnostic STO [27], International Classification of Diseases 11th Revision (ICD-11) [28], and Systematized Nomenclature of Medicine—Clinical Terms (SNOMED CT) [29], and we selected the STO for reuse due to its high similarity with atherosclerotic cerebrovascular disease. Most of them were monolingual. SNOMED CT and ICD contained some Chinese terms, showing their efficacy in term extension. In this step, the bilingual concepts were automatically extracted to be the counterpart terms in the ontology. Few Chinese ontologies were discovered to be useful; no ontologies were reused in the structure design. However, several Chinese terminologies or ontologies showed their effectiveness in term filling and detection. Among them, Chinese HPO [30] was partially reused to extend the manifestation branch. Physical medicine and rehabilitation terminology were used for term extraction and Chinese mapping in the rehabilitation branch. During the process of the ontology and terminology survey, no rehabilitation ontology and terminology that could be taken for reuse were found. However, rehabilitation was so important that it should not be discarded in atherosclerotic cerebrovascular disease ontology construction. Thus, we started from the beginning to establish the rehabilitation branch.

From the clinicians’ perspective, atherosclerotic cerebrovascular disease is a disease with high incidence without a clear boundary or unambiguous consensus. Thus, it is crucial to share the existing perception of this disease in an appropriate way, namely ontology. For the above investigation and presented necessity, we constructed a cross-lingual atherosclerotic cerebrovascular disease ontology including 10 top-level classes, respectively, clinical manifestation, comorbidity, complication, diagnosis, model of atherosclerotic cerebrovascular disease, pathogenesis, prevention, rehabilitation, risk factor, and treatment. The proposed ontology could provide well-organized knowledge of the disease and help researchers with retrieval and disease understanding. It could also support different applications in various scenarios, for instance, the new expressions discovery in electronic health records based on the existing ontology, named entity recognition and auto annotation with the ontology, and extension and integration of new triples or new knowledge graphs with the ontology as a basis. Since its cross-lingual properties, researchers from different countries, especially China, could quickly identify the counterpart expressions in another language and make a search strategy. The innovation gap of the ontology contained the scoping limitation of atherosclerotic cerebrovascular disease where there is no existing consensus but quite important, deep extension of phenotype concepts for disease identification, cross-lingual parallel corpus construction to help cross-lingual search, and the brand-new rehabilitation ontology of atherosclerotic cerebrovascular disease.

## Materials and methods

### Ontology development

After investigation, it was discovered that the Ontology Development 101 method is one of the most popular and mature ontology development methods. Compared to OBO Foundry methods, the Ontology Development 101 method shows more flexibility and less strictness. The Ontology Development 101 method contained seven steps, respectively, “determine the domain and scope, consider reusing existing ontologies, enumerate important terms, define the classes and the class hierarchy, define the properties of classes, define the facets of the slots, and create instances” [31]. We developed the ontology based on the combination of the Ontology Development 101 methodology [31] and expert opinions. The proposed ontology construction encompassed the following steps.

#### Step 1: Collect and specify the clinical requirements

In this step, we specify the clinical requirements and figure out what requirement is proposed, to what extent the established ontology could assist in solving the problem, what is the ontology targeting, and how to evaluate the effectiveness and necessity.

#### Step 2: The background investigation and knowledge acquisition

After investigating the related content in ontology modeling, medical ontology construction, and cross-lingual resources, we decided to develop a cross-lingual ontology focusing on atherosclerotic cerebrovascular disease based on the Ontology Development 101 methodology and the valuable experience of the multi-disciplinary experts. The established ontology should cover the principal aspects of the disease, especially the clinical manifestation since the diagnosis is mainly up to the patient’s symptoms.

#### Step 3: Ontology reuse

We investigated the existing ontology, analyzed the advantages and disadvantages, and decided to reuse the STO. The STO is an up-to-date ontology aiming at brain stroke and covers a wide range of biomedical concepts from multiple perspectives [23]. Considering that atherosclerotic cerebrovascular disease is a subset of the stroke category, reusing STO could, to a large extent, facilitate the inheritance and extension and provide convenience for international collaboration.

#### Step 4: Identify the scope

The STO did not share the same target as the proposed ontology. Therefore, the overall framework needs to be revised, including branch tailoring, concept cutting, addition, and clinician evaluation. The stroke types included ischemic stroke, hemorrhagic stroke, and transient ischemic attack. The types of “stroke” and the types of “atherosclerotic cerebrovascular disease” are not the same. Thus, it is necessary to tailor the appropriate scope from the STO. A neurosurgical clinician was invited to add content that belongs to atherosclerotic cerebrovascular disease or remove the content that does not belong to the scope of atherosclerotic cerebrovascular disease.

#### Step 5: Define the schema

After the discussion with clinicians, data scientists, and ontology experts, we decided to design 10 top-level classes, respectively, encompassing clinical manifestation, comorbidity, complication, diagnosis, model of atherosclerotic cerebrovascular disease, pathogenesis, prevention, rehabilitation, risk factor, and treatment. We added “rehabilitation” and “model of atherosclerotic cerebrovascular disease” as two of the top classes because rehabilitation is an indispensable part of atherosclerotic cerebrovascular disease regardless of severity. The therapeutic schedule should be decided according to the recognition of the specific disease model. During the investigation of rehabilitation-related ontology or terminology, no promising result emerged for rehabilitation reuse. However, the importance of rehabilitation in atherosclerotic cerebrovascular disease is self-evident and should not be ignored. Thus, we need to construct a rehabilitation ontology from the beginning. After collecting the authoritative guidelines in cross languages, we extracted and integrated the framework of rehabilitation content on each side and determined the final framework once discussed with two experts in medical informatics who had rich experience in ontology construction and real-world medical practice.

#### Step 6: Concept extraction

During the research process on existing rehabilitation resources, we discovered that less integrated rehabilitation terminology, ontology, or sub-branches could be reused, only several terms were found relevant. We investigated the domestic and international rehabilitation-related guidelines and literature, respectively, extracted both outlines and detail catalog, integrated the bilingual schema and content after discussion with two experts with medical informatics backgrounds, and recorded the concepts once approved. After the rehabilitation class was constructed, the concepts from various guidelines in different languages were extracted and integrated into the new version. We used the top-down and bottom-up methods in rehabilitation ontology construction to extract concepts and generate schema [32].

#### Step 7: Concept extension

Since we already obtained partial concepts, the concept extension involved several aspects. First, concepts in clinical manifestation need to be enlarged since the diagnosis depends heavily on clinical features. We investigated the existing ontology and terminology focused on clinical manifestation and selected HPO as our targeted ontology, extending the sub-branches in HPO to the proposed ontology. Second, the terms in the proposed ontology need to be expanded since they have abundant synonyms to be discovered. Specifically, we mapped each concept with the counterpart in SNOMED CT and ICD-11 and incorporated the extra synonyms in the proposed ontology. We assigned the appropriate synonyms for terms without mapping results in the counterpart language by a postgraduate with a medical informatics background.

#### Step 8: Ontology verification

The ontology was reviewed by four clinicians in the neurosurgery department who had received medical training and had annotation experience. If two experts showed different decisions, then the third expert was incorporated. If the third expert was unsure about some particular term, the fourth expert then intervened in the project. The audition was focused on several aspects including the term scope (whether one concept belongs to the scope of atherosclerotic cerebrovascular disease), term accuracy (whether the terms in both languages are the synonyms of the concept), spelling mistakes (whether one term has spelling mistakes), term duplication (whether one term occurred in inappropriate situations, for instance, a manifestation term occurred in both manifestation class and treatment class), and language error (for instance, whether a Chinese term is indicated as English one). The term duplication and language error were detected by the system TBench [33].

#### Step 9: Ontology evaluation

To evaluate the ontology, first, we did some statistics on the ontology, ranging from the number of concepts to the number of terms in Chinese and English in each class. Second, we performed a literature classification task on the proposed ontology. We extracted 600 literature abstracts focused on atherosclerotic cerebrovascular disease. The inclusion and exclusion criteria were as follows: [1] the theme of the article should be in the scope of atherosclerotic cerebrovascular disease; [2] the article should be written in English; and [3] only research articles would be included, while other types of literature, such as reviews and letters, would not be included. A classification task was performed in four categories including treatment, risk factor, pathogenesis, and rehabilitation. We would explore whether and how the proposed ontology could be helpful. We used four groups of methods to measure the result: (i) clustering (*K*-means) and ontology-only, (ii) Bayes [34] and Bayes&Ontology, (iii) Support Vector Machine (SVM) [35] and SVM&Ontology, and (iv) PubMedBERT model and PubMedBERTl&Ontology model. We calculated the final weight-precision, weight-recall, and weight-*F*1 scores toward each measurement. Group (i) was performed without manually annotated labels. In the clustering method, since the abstracts consisted of sentences, we adopted Sentence-BERT [36] to represent the content and used *K*-means [37] to realize automatic classification. The Sentence-BERT [36] is a sentence embedding representation model always used in matching text semantic similarity. Therefore, it is appropriate to adopt Sentence-BERT in downstream classification tasks. The *K*-means clustering algorithm is one of the most representative and popular clustering algorithms in unsupervised learning suitable for classification tasks when no labels were provided. In this case, we adopted Sentence-BERT to perform the vectorization of sentences in the abstracts. Afterward, *K*-means was applied for clustering. Then, we manually annotated the clustering center. In the ontology-only method, we first recognized the terminologies in the abstract with the ontology and then used their statistical data for automatic classification. In this case, we counted the number of terminologies in each category and automatically determined the category with the highest number. Afterward, we annotated 600 titles and abstracts in four categories and divided them into training datasets and test datasets. We also used Bayes and SVM in group (iii) and (iv) methods. In the PubMedBERT method, since the abstracts were medical literature, we adopted PubMedBERT [37] to complete the classification task. In the PubMedBERT&Ontology method, we used PubMedBERT [37] to vectorize the abstracts and to learn the parameters for classification. Meanwhile, we used the pair ontology corpus as the training dataset. For each targeted category, i.e. top-class in the ontology, the ontology terminology together with their top class, i.e. the category, would constitute a pair, and thus a pair ontology corpus was established. We used the corpus as the training data and PubMedBERT [37] as the exploring algorithm to find out whether the ontology would increase the classification performance. Considering that the distribution of the literature classification was different, we calculated the weight-precision, weight-recall, and weight-*F*1 scores to explore the results. The formula is listed below.

Assuming that the sample size is *n*, the sample size of category *i* is ${\text{Coun}}{{\text{t}}_i}$, and the precision, recall, and *F*1 score of the category *i* is ${P_i},{\ }{R_i},F{1_i}$:


$${\text{weighted - }}P = \sum\limits_{i \in 1, \ldots ,n} {W_i}*{P_i}$$

$${\text{weighted - }}R = \sum \limits_{i \in 1, \ldots ,n} {W_i}*{R_i}$$

$${\text{weighted - }}F1 = \sum \limits_{i \in 1, \ldots ,n} {W_i}*F{1_i}$$

$${W_i} = \frac{{{\text{Coun}}{{\text{t}}_i}}}{{{ \sum \nolimits}_{i \in 1, \ldots ,n}{\text{Coun}}{{\text{t}}_i}}} \\[14pt]$$

After obtaining the classification result, we carried out a further error analysis. The weight-precision, weight-recall, and weight-*F*1 scores of each category were calculated.

### Ethical approval

The study does not involve experiments on humans or animals. Thus, the ethics committee approval was not required.

## Results

### Hierarchical structure

**Figure 1.**
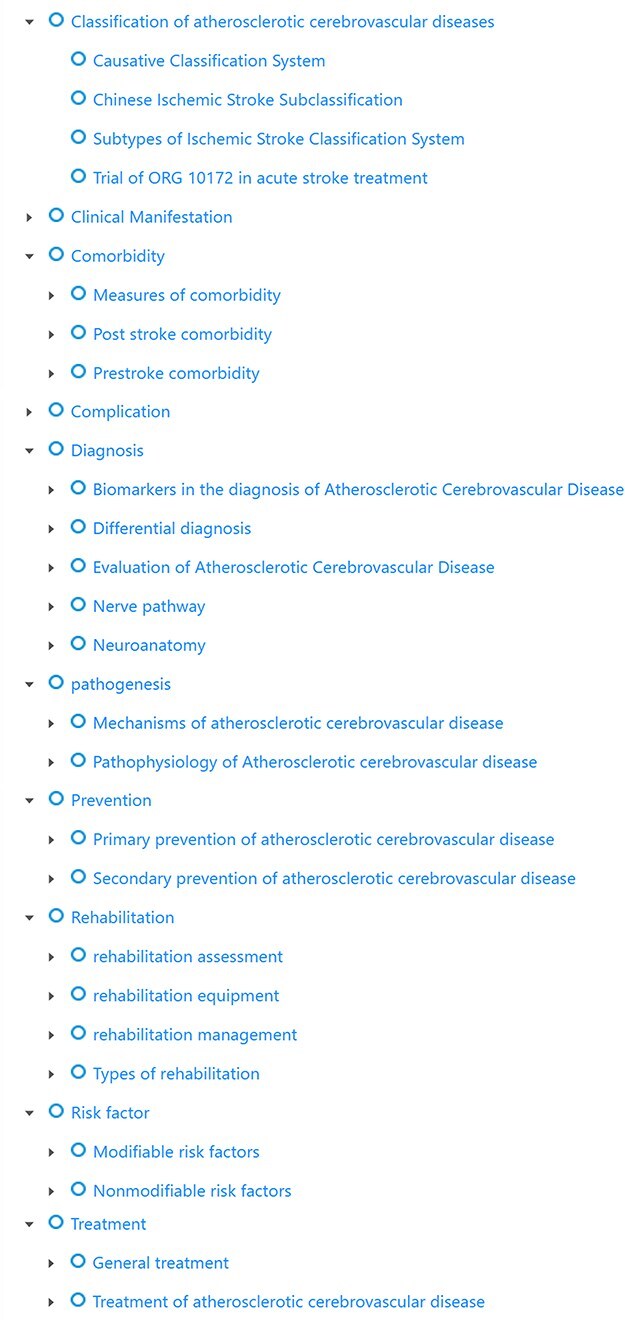
The hierarchical structure. The ontology was classified into 10 categories: clinical manifestation, comorbidity, complication, diagnosis, model of atherosclerotic cerebrovascular disease, prevention, rehabilitation, risk factor, and treatment.

The proposed ontology covered the core concepts in atherosclerotic cerebrovascular disease, which can help clinicians and physicians realize cross-lingual retrieval, assist researchers in the field to define the scope in a short time, and quickly understand the disease knowledge framework. It could also be used in applications such as data mining and named entity recognition. The current ontology included 10 top-level classes, respectively, clinical manifestation, comorbidity, complication, diagnosis, model of atherosclerotic cerebrovascular disease, pathogenesis, prevention, rehabilitation, risk factor, and treatment. The clinical manifestation class contained different symptoms according to various syndromes, for instance, the anterior cerebral artery infarction syndrome encompassed akinetic mutism and behavioral disturbance. The comorbidity class contained the measures of comorbidity, the post-stroke of comorbidity, and the prestrike comorbidity. The complication class included the complications that occurred during atherosclerotic cerebrovascular disease, such as cardiac arrest and dysphagia. The diagnosis class encompassed biomarkers in the diagnosis, diagnosis in brain anatomy, differential diagnosis, evaluation of atherosclerotic cerebrovascular disease, and nerve pathway. The model of the atherosclerotic cerebrovascular disease class included several types of the disease, for instance, Trial of ORG 10172 in Acute Stroke Treatment and Causative Classification of Stroke System. The pathogenesis contained the mechanisms and the pathophysiology of atherosclerotic cerebrovascular disease. The prevention class involved primary prevention such as dietary habits and education, and the secondary prevention ranged from drug therapy to lifestyle modification. The risk factor class introduced two types of factors: modifiable risk factors such as dietary factors and nonmodifiable risk factors such as gender and age. The treatment class was divided into two parts, the general treatment and the treatment of atherosclerotic cerebrovascular disease. The rehabilitation class as the highlighted branch is a novelty in the current ontology, which contains the assessment, equipment, management, and types. Figure 1 shows the hierarchical structure of the ontology.

### Rehabilitation design

**Figure 2.**
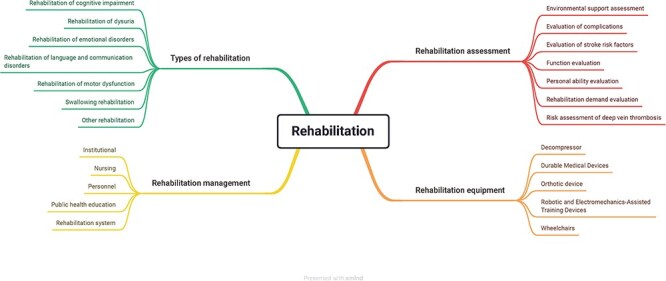
The rehabilitation ontology design. The rehabilitation ontology comprised types of rehabilitation, rehabilitation management, rehabilitation assessment, and rehabilitation equipment.

The rehabilitation ontology was divided into four sessions: types, management, assessment, and equipment. The types of rehabilitation included various categories of rehabilitation, e.g. cognitive impairment and rehabilitation of dysuria. The rehabilitation management encompassed the institution, nursing, personnel, public health education, and rehabilitation system. The rehabilitation assessment contained the factors that could influence the rehabilitation results, for instance, environmental support, complications, function, and personal ability. The equipment covered the current common device used in rehabilitation. With the upgrading of technologies, the equipment concepts should also be updated in the ontology. Figure 2 shows the detailed information of rehabilitation design.

### Ontology statistics

**Figure 3.**
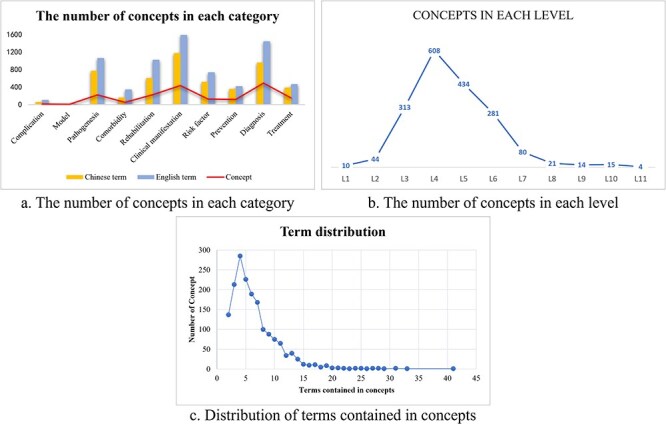
The ontology statistics on concepts. (a) The numbers of English and Chinese terms in each category were shown, together with the number of total concepts. (b) The concepts in Level 4 reached the most among all levels. (c) One concept could comprise multiple terms. Nearly 300 concepts contain about five synonyms. Concept with the most synonyms reached >40 terms.

We carried out the data statistics of the proposed ontology, Table 1 shows the overview of the ontology including the number of concepts, the top-level classes, the number of English/Chinese terms, the number of English/Chinese synonyms, and the number of English/Chinese definitions in each topic. There are a total of 1715 concepts in the ontology, covering 4588 Chinese terms, 6617 English terms, and 972 definitions. Clinical manifestations class and diagnosis class contained the most concepts and accounted for >50% of the total. Among them, clinical manifestations showed the most Chinese and English synonyms, 742 and 1152, respectively, and the most definitions with 173 Chinese definitions and 373 English definitions. The rehabilitation ranked third with 224 concepts, assigning its Chinese and English terms as 603 and 1022, together with 25 Chinese definitions. As a completely new branch, rehabilitation played an indispensable role whether in ontology or the real-world therapy pathway. The model of atherosclerotic cerebrovascular disease contained the least concepts, only five concepts were included. Among all the concepts, 23 contained >20 synonyms, up to 41 at most, and 309 contained >10 synonyms. The proposed ontology consists of many levels, with the deepest level reaching 11, which is presented in the pathogenesis class. Over 60% of the top-level classes contained eight levels, and most concepts could be discovered in Level 4. Figure 3 shows the ontology statistics on the number of concepts. Figure 3c demonstrates the distribution of terms included in concepts, in which ∼300 concepts contained five synonyms, the most synonyms reached up to 41 in one concept.

**Table 1.** The ontology statistics

Concept branch	Concept	Chinese term	English term	Chinese synonyms	English synonyms	Chinese definition	English definition	Level
Diagnosis	488	955	1442	467	953	8	219	8
Clinical manifestation	431	1173	1583	742	1152	173	373	9
Rehabilitation	224	603	1022	379	798	25	0	6
Pathogenesis	219	769	1059	550	840	0	78	11
Treatment	150	385	463	235	313	0	53	8
Risk factor	126	516	735	390	609	0	32	2
Prevention	117	355	417	238	300	0	54	8
Comorbidity	51	160	341	109	290	0	29	7
Complication	13	55	103	42	90	2	7	3
Model	5	6	18	1	13	0	2	2
Total (deduplication)	1715	4588	6617	2873	4901	198	774	11

### Ontology evaluation

According to the prediction result, the ontology could prove its usefulness in multiple aspects. Compared to the PubMedBERT model, the PubMedBERT&Ontology could increase the weight-precision from 81.74% to 82.52%, the weight-recall from 78.15% to 79.47%, and the weight-*F*1 score from 79.19% to 80.30%. The ontology as a supplementary training corpus would be a considerable way to utilize it for classification tasks. Compared to the SVM method, the SVM&Ontology method could enhance the weight-precision from 77.42% to 81.54%, weight-recall from 77.48% to 78.15%, and weight-*F*1 from 76.63% to 78.97%. The Bayes method demonstrated the weight-*F*1 as 70.60%, and the counterpart method Bayes&Ontology increased the result to 76.19%, almost an 8% increase. All the comparison results showed the effectiveness of the ontology in classification tasks and insight into how to use the ontology. With no labels provided, the ontology-only method illustrated nearly 24% higher accuracy than *K*-means. It proved that ontology is indeed useful for downstream tasks. The exact scores are listed in Table 2.

**Table 2.** The evaluation results of text classification

Methods	Weight-precision	Weight-recall	Weight-*F*1
Clustering (*K*-means)	62.32	40.40	45.18
Ontology-only	57.00	57.62	55.91
Bayes	79.24	67.55	70.60
Bayes&Ontology	78.43	75.50	76.19
SVM	77.42	77.48	76.63
SVM&Ontology	8154	78.15	78.97
PubMedBERT	81.74	78.15	79.19
PubMedBERT&Ontology	82.52	79.47	80.30

In the error analysis, the weight-*F*1 score performed the highest in the category “treatment,” where 84 test samples were in the test dataset, also ranked first. On the contrary, the weight-*F*1 score performed the lowest in the category “rehabilitation,” where the test dataset contained the least eight test samples. It indicated that the PubMedBERT&Ontology method showed higher capability in identifying categories with higher quantities of test datasets. However, it performed poorer fitting ability for those with scarce samples. A sample extension might improve its effectiveness. Errors in classification tasks may be due to scarce test samples, deficient training samples, less ontology comprehensiveness, inadequate ontology vocabulary, and insufficient model fitting ability. Table 3 shows the error analysis statistics of classification results for PubMedBERT&Ontology.

**Table 3.** Error analysis of classification results for PubMedBERT&Ontology

Category	Weight-precision	Weight-recall	Weight-*F*1 score	No. of samples in the test dataset
Treatment	0.9437	0.7976	0.8645	84
Risk factor	0.7451	0.8085	0.7755	47
Pathogenesis	0.5263	0.8333	0.6452	12
Rehabilitation	0.5000	0.6250	0.5556	8
Weight-average	0.8252	0.7947	0.8030	151

### Named entity recognition

**Figure 4.**
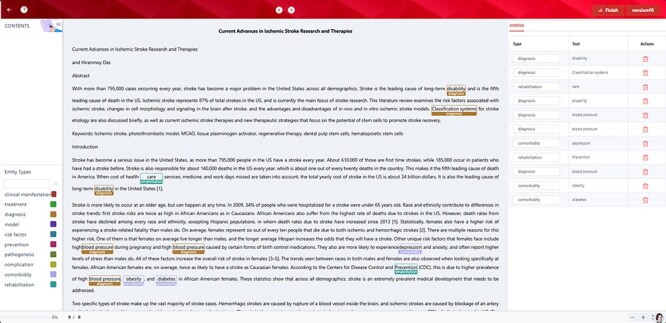
Named entity recognition by the ontology. Ten categories in the proposed ontology were used to auto-annotate one abstract related to atherosclerotic cerebrovascular disease. Among them, depression, obesity, and diabetes were annotated as comorbidity.

The ontology contained the concepts in atherosclerotic cerebrovascular disease, covering abundant bilingual synonyms in the field, and thus, it could help realize the auto-annotation to accomplish named entity recognition. We imported the proposed ontology in Comma, a cross-lingual medical text annotation platform [38], as the embedded lexicon so that the users could take advantage of it any time without customizing the ontology as one uploaded lexicon with the required format. The auto-annotation results could support the following data training and corpus forming, which would sharply reduce the cost and time of data foundations. Meanwhile, the named entity recognition provided by the proposed ontology would, to a large extent, assist physicians with phenotypic recognition, diagnosis checking, treatment retrieval, and effect recognition thereby supporting clinical decision-making. Figure 4 shows the named entity recognition annotated by the proposed ontology.

### Knowledge fusion

**Figure 5.**
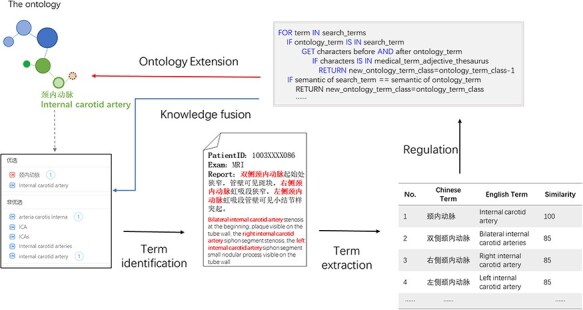
Knowledge fusion case based on the ontology. Partial electronic health record information was extracted through models combined with the ontology such as “bilateral internal carotid artery.” The extracted expression was compared with the ontology based on similarity; the expression with higher similarity that did not exist in the ontology would be used for ontology extension, thus realizing knowledge fusion.

Plenty of new terms or new expressions were produced in real-world medical records. Based on the proposed ontology, we could identify entities in medical records through entity recognition algorithms, detect novel expressions based on the proposed ontology, rules, and similarity detection algorithms, and integrate the expressions to extend the ontology and realize novel expressions detection. We implemented a particular case to realize ontology validation to check if it could support clinical use. In this case, we used a branch part in the proposed ontology, i.e. the brain structure branch ontology, to realize term identification and discover the novel expressions in the real-world electronic health record and obtained several candidate expressions toward one concept. The similarity demonstrated the relevancy of the concept and candidates. Specific rules were made to remind the term addition and the location where it should be added. In this case, the new expressions ‘右侧颈内动脉(right internal carotid artery)’ and ‘左侧颈内动脉(left internal carotid artery)’ should be extended as children nodes of ‘颈内动脉(internal carotid artery)’. Thus, the knowledge fusion could be implemented, and the ontology could be extended. Figure 5 shows the demo of knowledge fusion case.

## Discussion

### Principal results

Ontology targeting a specific field plays an important role in knowledge standardization, integration, annotation, and representation and could assist clinical decision support. An earlier study presented an ontology focused on adverse drug reactions as a machine-understandable foundation for medical informatics analysis and other clinical decision support [39]. Another study constructed a cancer ontology covering consumer concepts to realize social media analysis and to provide support to related health requirements [40]. A depression ontology was established for social media data analysis and logic evaluation to back up sentimental analysis application [41]. A fertility-related ontology was demonstrated and could be considered as the basic framework for future fertility signals detection [42]. The development of an ontology could help model multisource heterogeneous health data and generate personalized suggestions based on rules setting and semantic annotation [43].

As a disease with high incidence, there is not any corresponding ontology toward atherosclerotic cerebrovascular disease that could allow researchers to obtain instant concept acquisition. In addition, there is no clear boundary and consensus on the specific disease worldwide. Therefore, we cooperated with the top hospital and developed a highly structured and relatively comprehensive ontology toward atherosclerotic cerebrovascular disease through concept extraction and hierarchy construction as a reference for researchers, experts, and clinicians in this field. The current ontology mainly consists of 1715 concepts, 4588 Chinese terms, and 6617 English terms. Its highlights included the deep extension according to the clinical requirement, the ontology design on rehabilitation, and cross-lingual term content. The ontology could assist in entity recognition and sequentially help with the clinical diagnosis and treatment. However, one symptom or several symptoms could not perfectly and accurately conclude some patients suffered from atherosclerotic cerebrovascular disease. More related work should be implemented, for example, how to combine ontology identification and deep learning models and implement cross-verification and evaluation to provide more trustworthy suggestions. The potential applications based on the proposed ontology are listed below.

### Application

**Figure 6.**
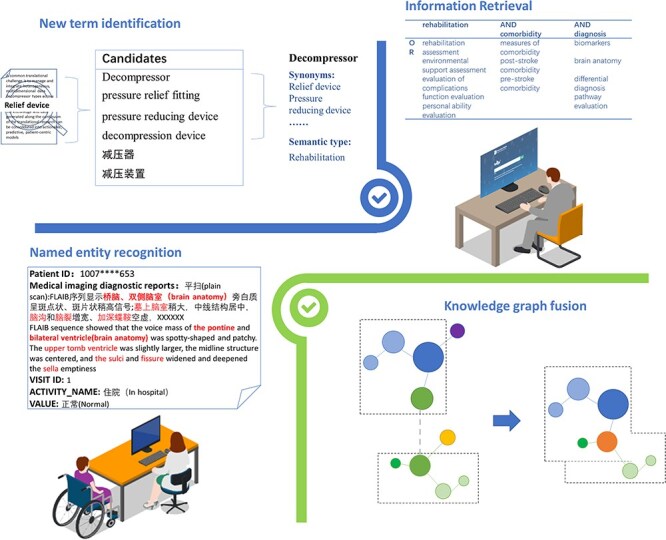
The application and scenario. The proposed ontology could be used for new term identification, information retrieval, named entity recognition, and knowledge graph fusion.

The ontology could provide support for various scenarios including cross-lingual information retrieval for related knowledge, named entity recognition for following training, novel expression discovery in real-world data through similarity matching algorithms, and knowledge fusion to form a larger and more comprehensive knowledge graph. In addition, when integrating with large language models (LLMs), ontology could augment LLMs as structured knowledge representations to better classify and structure domain-specific knowledge into top-level concepts [44]; enhance LLMs’ reasoning capabilities by generating logical relationships [45]; reduce bias and improve accuracy in LLMs by providing a reliable knowledge base [46]; and improve interpretability of LLMs by offering clear structures for knowledge [47]. Figure 6 shows the application and scenario of the proposed ontology.

#### Cross-lingual scenarios

Ontologies could naturally assist literature retrieval through expanded concepts and a more accurate range. A childhood vaccination ontology was evaluated to be useful for assisting in the identification of the emotions of children experiencing the vaccine [48]. An ontology-based care knowledge platform was published for information retrieval and inquiry, and thus references would be provided and better care could be served [49]. A physical activity ontology was established for knowledge retrieval and information extraction so that data structuring and standardization could be feasible [50]. Since there is no commonly acknowledged consensus on atherosclerotic cerebrovascular disease, physicians, medical informatics experts, and researchers in this field would need to investigate more knowledge and frontier achievements, especially in literature. The ontology provided such scope for the disease. The ontology could help clinicians quickly understand the scope of the disease and assist in the patient’s situation such as diagnosis. Lopsided information resources in disparate languages could lead to differentiated knowledge distribution, and the cross-lingual demand for clinicians could not be ignored [51, 52].

Cross-lingual ontologies are crucial for bridging language barriers in semantic web applications, enabling knowledge sharing and interoperability across different languages. A cross-lingual ontology could support cross-lingual named entity recognition [53], cross-lingual information retrieval [54], cross-lingual text mining [55], multilingual model transfer learning [56], cross-lingual ontology mapping [57], and zero-shot cross-lingual transfer [58]. Table 4 lists the concrete tasks under cross-lingual retrieval scenarios, the roles of cross-lingual ontology, and related cases.

**Table 4.** Application in cross-lingual scenarios

Tasks	What can cross-lingual ontology do?	Cases
Cross-lingual named entity recognition (NER)	Identify and link the same entities in different languages, thus promoting the multilingual knowledge base construction and information retrieval	Proposed methods using bilingual word embeddings and self-attention mechanisms to enhance cross-lingual NER [53]
Cross-lingual information retrieval (CLIR)	Help understand queries and documents in different languages, thereby improving retrieval accuracy	Models such as BERT are fine-tuned for CLIR to learn the relevance between English queries and documents in other languages [54]
Cross-lingual text mining	Assist in extracting valuable information from texts across languages	Automatically mining cross-lingual entity pairs enriches multilingual entity dictionaries, beneficial for machine translation [55]
Multilingual model transfer learning	Facilitate the transfer of models from one language to another, especially important for low-resource languages	Methods using adversarial networks and mixture-of-experts models to learn language-invariant features and leverage language similarities [56]
Cross-lingual ontology mapping	Aligning ontologies across languages through knowledge sharing and data integration	Proposed strategies to enhance mapping accuracy [57]
Zero-shot cross-lingual transfer	Learning joint sentence representations across many languages allows model transfer without annotated data in the target language	Effective in tasks such as cross-lingual natural language inference, document classification, and parallel corpus mining [58]

#### Ontology reuse cases

In this section, different ontology reuse cases were presented. We listed applications on how to utilize ontologies. Apart from basic tasks such as information retrieval, automatic annotation, and knowledge extraction, ontology could provide clinical decision support [59–61], promote medical knowledge organization and data sharing [43, 62, 63], allow medical data processing and mining [64–66], and facilitate downstream tasks such as classification or categorization [67]. Detailed information is listed in Table 5.

**Table 5.** Ontology-based applications and reuse cases

Applications	Reuse cases	Cases
Clinical decision support systems (CDSS)	Help enhance decision-making by providing structured clinical knowledge and evidence-based guidelines	To realize fatty liver detection based on ontology and detect rules from the decision tree algorithm [59]
		Use ontology to replace databases to generate an ontology-based CDSS to reduce medication errors [60]
		Applied ontology into the generation of CBT action plans for treating mild depression [61]
Medical knowledge organization and data sharing	Help researchers organize and retrieve relevant biological and medical information efficiently	Provide standardized and hierarchical ontology of prostate cancer for future knowledge graph extraction, deep phenotyping, and explainable artificial intelligence development [62]
	Standardize the terminology across various Electronic Health Record systems, making data interoperable across different healthcare facilities	Develop an ontology to annotate data from heterogeneous sources and standardize the descriptions to generate recommendations [43]
		Generate an automatic ontology-based data integration method by semantic integration of heterogeneous data [63]
Medical data processing and mining	Increase the efficiency of information retrieval	Propose an ontology-based semantic model for query expansion to realize efficient information retrieval [64]
	Achieve deep representation of clinical text	Improve the characterization of clinical text by utilizing the integration of ontologies [65]
		Realize drug class effect analysis through ontology-based deep representation [66]
Classification	Categorize clinical studies	Apply ontology to classify the clinical studies [67]

### Limitations

The current ontology is still not comprehensive enough to contain all the information that occurred in this field and lacks some relationships that could be helpful in further applications. In addition, limited ontology applications were provided. The evaluation of ontology applications such as cross-lingual scenarios was not carried out. More explorations should be carried out in the future.

## Conclusions

As a disease with high health hazards and popular incidence, atherosclerotic cerebrovascular disease has not obtained enough attention. Up till now, no systematic concept datasets have been released to help clinicians in the field to clarify the scope, assist in research, and offer maximized value. The proposed ontology aims to solve the above scientific problems by providing a clear set of cross-lingual concepts and terms with an explicit hierarchical structure, helping scientific researchers to quickly retrieve relevant medical literature, assisting data scientists to efficiently identify relevant contents in electronic health records, and providing a clear domain framework for academic reference. The ontology is now released at BioPortal for public use [68].

## Data Availability

The ontology is now available at https://bioportal.bioontology.org/ontologies/ACVD_ONTOLOGY.

## References

[1] Tsantilas P , Lao S, Wu Z et al. Chitinase 3 like 1 is a regulator of smooth muscle cell physiology and atherosclerotic lesion stability. *Cardiovasc Res* 2021;117:2767–80. doi: 10.1093/cvr/cvab014 33471078 PMC8848327

[2] Lee SC , Son KJ, Hoon Han C et al. Cardiovascular and cerebrovascular-associated mortality in patients with preceding bronchiectasis exacerbation. *Ther Adv Respir Dis* 2022;16:17534666221144206. doi: 10.1177/17534666221144206 PMC9772950 36533883

[3] Wang W , Jiang B, Sun H et al. Prevalence, incidence, and mortality of stroke in China: results from a nationwide population-based survey of 480 687 adults. *Circulation* 2017;135:759–71. doi: 10.1161/CIRCULATIONAHA.116.025250 28052979

[4] Liu L , Chen W, Zhou H et al. Chinese Stroke Association guidelines for clinical management of cerebrovascular disorders: executive summary and 2019 update of clinical management of ischaemic cerebrovascular diseases. *Stroke Vasc Neurol* 2020;5:159–76. doi: 10.1136/svn-2020-000378 32561535 PMC7337371

[5] Barthels D , Das H. Current advances in ischemic stroke research and therapies. *Biochim Biophys Acta Mol Basis Dis* 2020;1866:165260. doi: 10.1016/j.bbadis.2018.09.012 PMC6981280 31699365

[6] Virani SS , Alonso A, Benjamin EJ et al. Heart disease and stroke statistics-2020 update: a report from the American Heart Association. *Circulation* 2020;141:e139–596. 31992061 10.1161/CIR.0000000000000757

[7] Saini V , Guada L, Yavagal DR. Global epidemiology of stroke and access to acute ischemic stroke interventions. *Neurology* 2021;97:S6–16. doi: 10.1212/WNL.0000000000012781 34785599

[8] Khoshnam SE , Winlow W, Farzaneh M et al. Pathogenic mechanisms following ischemic stroke. *Neurol Sci* 2017;38:1167–86. 28417216 10.1007/s10072-017-2938-1

[9] Tsuchiya M , Sako K, Yura S et al. Cerebral blood flow and histopathological changes following permanent bilateral carotid artery ligation in Wistar rats. *Exp Brain Res* 1992;89:87–92. doi: 10.1007/BF00229004 1601104

[10] Johnson CO , Nguyen M, Roth GA. Global, regional, and national burden of stroke, 1990-2016: a systematic analysis for the Global Burden of Disease Study 2016. *Lancet Neurol* 2019;18:439–58. doi: 10.1016/S1474-4422(19)30034-1 30871944 PMC6494974

[11] Safarova MS , Kullo IJ. Using the electronic health record for genomics research. *Curr Opin Lipidol* 2020;31:85–93. doi: 10.1097/MOL.0000000000000662 32073412 PMC9229554

[12] Wang J , Bai L, Shi M et al. Trends in age of first-ever stroke following increased incidence and life expectancy in a low-income Chinese population. *Stroke* 2016;47:929–35. doi: 10.1161/STROKEAHA.115.012466 26869385

[13] Krishnamurthi RV , Feigin VL, Forouzanfar MH et al. Global and regional burden of first-ever ischaemic and haemorrhagic stroke during 1990-2010: findings from the Global Burden of Disease Study 2010. *Lancet Glob Health* 2013;1:e259–281. doi: 10.1016/S2214-109X(13)70089-5 25104492 PMC4181351

[14] Winstein CJ , Stein J, Arena R et al. Guidelines for adult stroke rehabilitation and recovery: a guideline for healthcare professionals from the American Heart Association/American Stroke Association. *Stroke* 2016;47:e98–169. 27145936 10.1161/STR.0000000000000098

[15] Stinear CM , Lang CE, Zeiler S et al. Advances and challenges in stroke rehabilitation. *Lancet Neurol* 2020;19:348–60. doi: 10.1016/S1474-4422(19)30415-6 32004440

[16] Chang WH , Shin YI, Lee SG et al. Characteristics of inpatient care and rehabilitation for acute first-ever stroke patients. *Yonsei Med J* 2015;56:262–70. doi: 10.3349/ymj.2015.56.1.262 25510773 PMC4276765

[17] Kim WS , Bae HJ, Lee HH et al. Status of rehabilitation after ischemic stroke: a Korean nationwide study. *Ann Rehabil Med* 2018;42:528–35. doi: 10.5535/arm.2018.42.4.528 30180521 PMC6129701

[18] Musicco M , Emberti L, Nappi G et al. Early and long-term outcome of rehabilitation in stroke patients: the role of patient characteristics, time of initiation, and duration of interventions. *Arch Phys Med Rehabil* 2003;84:551–58. doi: 10.1053/apmr.2003.50084 12690594

[19] Paolucci S , Antonucci G, Grasso MG et al. Early versus delayed inpatient stroke rehabilitation: a matched comparison conducted in Italy. *Arch Phys Med Rehabil* 2000;81:695–700. doi: 10.1016/S0003-9993(00)90095-9 10857508

[20] Toschke AM , Tilling K, Cox AM et al. Patient-specific recovery patterns over time measured by dependence in activities of daily living after stroke and post-stroke care: the South London Stroke Register (SLSR). *Eur J Neurol* 2010;17:219–25. doi: 10.1111/j.1468-1331.2009.02774.x 19682061

[21] Gruber TR . Toward principles for the design of ontologies used for knowledge sharing? *Inter J Human Comp Stud* 1995;43:907–28. doi: 10.1006/ijhc.1995.1081

[22] Reyes-Peña C , Tovar M, Bravo M et al. An ontology network for Diabetes Mellitus in Mexico. *J Biomed Semantics* 2021;12:19. doi: 10.1186/s13326-021-00252-2 PMC8500829 34625104

[23] Habibi-koolaee M , Shahmoradi L, Niakan Kalhori SR et al. STO: stroke ontology for accelerating translational stroke research. *Neurol Ther* 2021;10:321–33. doi: 10.1007/s40120-021-00248-1 33886080 PMC8140017

[24] Jensen M , Cox AP, Chaudhry N et al. The neurological disease ontology. *J Biomed Semantics* 2013;4:42. doi: 10.1186/2041-1480-4-42 PMC4028878 24314207

[25] Symptom Ontology. 2023. https://bioportal.bioontology.org/ontologies/SYMP (5 November 2024, date last accessed).

[26] Köhler S , Gargano M, Matentzoglu N et al. The human phenotype ontology in 2021. *Nucleic Acids Res* 2021;49:D1207–17. doi: 10.1093/nar/gkaa1043 33264411 PMC7778952

[27] Podsiadly-Marczykowska T Ciszek B Przelaskowski A . Development of diagnostic stroke ontology - preliminary results. In: Piętka E, Kawa J, Wieclawek W (eds), *Information Technologies in Biomedicine*. Vol. 4. Cham: Springer International Publishing, 2014, 261–72

[28] International Classification of Diseases for Mortality and Morbidity Statistics. 2022. https://icdcdn.who.int/icd11referenceguide/en/html/index.html (5 November 2024, date last accessed).

[29] SNOMED CT. 2023. https://www.nlm.nih.gov/healthit/snomedct/snomed_overview.html (5 November 2024, date last accessed).

[30] Chinese Human Phenotype Ontology. 2024. https://www.chinahpo.net (5 November 2024, date last accessed).

[31] Noy N . Ontology Development 101: A Guide to Creating Your First Ontology. 2001.

[32] Francesconi E Montemagni S Peters W et al. Integrating a bottom-up and top-down methodology for building semantic resources for the multilingual legal domain. Vol. 6036. In: Francesconi E, Montemagni S, Peters W, Tiscornia D (eds.), *Semantic Processing of Legal Texts*. *Lecture Notes in Computer Science*. Berlin, Heidelberg, Germany: Springer, 2010, 95–121.

[33] Deng P , Ji Y, Shen L et al. TBench: A collaborative work platform for multilingual terminology editing and development. *Stud Health Technol Inform* 2019;264:1449–50. 31438175 10.3233/SHTI190478

[34] Duda RO , Hart PE (eds). *Pattern Classification and Scene Analysis*. Menlo Park, CA: Wiley Interscience, 1974.

[35] Cortes C , Vapnik V. Support-vector networks. *Machine Learning* 1995;20:273–97. doi: 10.1007/BF00994018

[36] Reimers N , Gurevych I. *Sentence-BERT: Sentence Embeddings Using Siamese BERT-Networks*. Hong Kong: Association for Computational Linguistics, 2019, 3982–92.

[37] MacQueen J . Some Methods for Classification and Analysis of Multivariate Observations. 1967.

[38] Comma. https://comma.phoc.org.cn/ (5 November 2024, date last accessed).

[39] Li X , Lin X, Ren H et al. Ontological organization and bioinformatic analysis of adverse drug reactions from package inserts: development and usability study. *J Med Internet Res* 2020;22:e20443. doi: 10.2196/20443 PMC7400033 32706718

[40] Lee J , Park H-A, Park SK et al. Using social media data to understand consumers’ information needs and emotions regarding cancer: ontology-based data analysis study. *J Med Internet Res* 2020;22:e18767. doi: 10.2196/18767 PMC7752532 33284127

[41] Jung H , Park H-A, Song T-M. Ontology-based approach to social data sentiment analysis: detection of adolescent depression signals. *J Med Internet Res* 2017;19:e259. doi: 10.2196/jmir.7452 PMC5547245 28739560

[42] Lee J-H , Park H-A, Song T-M. A determinants-of-fertility ontology for detecting future signals of fertility issues from social media data: development of an ontology. *J Med Internet Res* 2021;23:e25028. doi: 10.2196/25028 PMC8240803 34125068

[43] Chatterjee A , Prinz A, Gerdes M et al. An automatic ontology-based approach to support logical representation of observable and measurable data for healthy lifestyle management: proof-of-concept study. *J Med Internet Res* 2021;23:e24656. doi: 10.2196/24656 PMC8065560 33835031

[44] Mateiu P , Groza A. Ontology engineering with large language models. In: *2023 25th International Symposium on Symbolic and Numeric Algorithms for Scientific Computing*. Nancy, France: IEEE, 2023, 226–229.

[45] Toro S , Anagnostopoulos AV, Bello S et al. Dynamic Retrieval Augmented Generation of Ontologies using Artificial Intelligence (DRAGON-AI). *J Biomed Semant* 2024;15:19. doi: 10.1186/s13326-024-00320-3 PMC11484368 39415214

[46] Neuhaus F . Ontologies in the era of large language models - a perspective. *Appl Ontol* 2023;18:399–407. doi: 10.3233/AO-230072

[47] Shimizu C , Hammar K, Hitzler P. Modular ontology modeling. *Semant Web* 2022;14:459–89. doi: 10.3233/SW-222886

[48] On J , Park H-A, Song T-M. Sentiment analysis of social media on childhood vaccination: development of an ontology. *J Med Internet Res* 2019;21:e13456. doi: 10.2196/13456 PMC6592483 31199290

[49] Kim G , Jeon H, Park SK et al. A care knowledge management system based on an ontological model of caring for people with dementia: knowledge representation and development study. *J Med Internet Res* 2021;23:e25968. doi: 10.2196/25968 PMC8262671 34100762

[50] Kim H , Mentzer J, Taira R. Developing a physical activity ontology to support the interoperability of physical activity data. *J Med Internet Res* 2019;21:e12776. doi: 10.2196/12776 PMC6658272 31012864

[51] Ma H , Yang F, Ren J et al. ECCParaCorp: a cross-lingual parallel corpus towards cancer education, dissemination and application. *BMC Med Inform Decis Mak* 2020;20:122. doi: 10.1186/s12911-020-1116-1 PMC7346326 32646415

[52] Jin H , Li C, Zhang J et al. XLORE2: Large-scale cross-lingual knowledge graph construction and application. *Data Intell* 2019;1:77–98. doi: 10.1162/dint_a_00003

[53] Zhang X , Yu B, Cao J et al. Representation and labeling gap bridging for cross-lingual named entity recognition. *Proceedings of the 46th International ACM SIGIR Conference on Research and Development in Information Retrieval*, pp. 1230–40. Taipei: Association for Computing Machinery, 2023.

[54] Jiang Z , El-Jaroudi A, Hartmann W et al. Cross-lingual information retrieval with BERT. *Cross-Language Search and Summarization of Text and Speech (CLSSTS2020)*, May 2020. Marseille, France: European Language Resources Association, 2020, 26–31.

[55] El-Kishky A , Renduchintala A, Cross J et al. XLEnt: mining a large cross-lingual entity dataset with lexical-semantic-phonetic word alignment. *Proceedings of the 2021 Conference on Empirical Methods in Natural Language Processing*, November 2021. Online and Punta Cana, Dominican Republic: Association for Computational Linguistics , 2021, 10424–10430.

[56] Chen X , Awadallah AH, Hassan H et al. Multi-source cross-lingual model transfer: learning what to share. *Proceedings of the 57th Annual Meeting of the Association for Computational Linguistics*, July 2019. Florence, Italy: Association for Computational Linguistics, 2019, 3098–3112.

[57] Fu B , Brennan R, O’Sullivan D. Cross-Lingual Ontology Mapping – An Investigation of the Impact of Machine Translation. Berlin, Heidelberg: Springer, 2009, 1–15.

[58] Artetxe M , Schwenk H. Massively multilingual sentence embeddings for zero-shot cross-lingual transfer and beyond. *Trans Assoc Comput Ling* 2019;7:597–610. doi: 10.1162/tacl_a_00288

[59] Banihashem SY , Shishehchi S. Ontology-based decision tree model for prediction of fatty liver diseases. *Comput Methods Biomech Biomed Engin* 2023;26:639–49. doi: 10.1080/10255842.2022.2081502 35635206

[60] Calvo-Cidoncha E , Camacho-Hernando C, Feu F et al. OntoPharma: ontology based clinical decision support system to reduce medication prescribing errors. *BMC Med Inform Decis Mak* 2022;22:238. doi: 10.1186/s12911-022-01979-3 PMC9463735 36088328

[61] Nair A , Abidi SSR, Van Woensel W et al. Ontology-based personalized cognitive behavioural plans for patients with mild depression. *Stud Health Technol Inform* 2021;281:729–33. 34042672 10.3233/SHTI210268

[62] Yu C , Zong H, Chen Y et al. PCAO2: an ontology for integration of prostate cancer associated genotypic, phenotypic and lifestyle data. *Briefings Bioinf* 2024;25:bbae136. doi: 10.1093/bib/bbae136 PMC10982949 38557678

[63] Thirumahal R , Sudha Sadasivam G, Shruti P. Semantic integration of heterogeneous data sources using ontology-based domain knowledge modeling for early detection of COVID-19. *SN Comput Sci* 2022;3:428. doi: 10.1007/s42979-022-01298-4 PMC9362348 35965952

[64] Kumar R , Sharma SC. Hybrid optimization and ontology-based semantic model for efficient text-based information retrieval. *J Supercomput* 2023;79:2251–80. doi: 10.1007/s11227-022-04708-9 35967462 PMC9364863

[65] Slater LT , Bradlow W, Ball S et al. Improved characterisation of clinical text through ontology-based vocabulary expansion. *J Biomed Semantics* 2021;12:7. doi: 10.1186/s13326-021-00241-5 PMC8042947 33845909

[66] Wang L , Li M, Xie J et al. Ontology-based systematical representation and drug class effect analysis of package insert-reported adverse events associated with cardiovascular drugs used in China. *Sci Rep* 2017;7:13819. doi: 10.1038/s41598-017-12580-4 PMC5653862 29061976

[67] Liu H , Carini S, Chen Z et al. Ontology-based categorization of clinical studies by their conditions. *J Biomed Inform* 2022;135:104235. doi: 10.1016/j.jbi.2022.104235 36283581

[68] ACVD Ontology. https://bioportal.bioontology.org/ontologies/ACVD_ONTOLOGY (5 November 2024, date last accessed).

